# Genetic Reassessment Reveals Catecholaminergic Polymorphic Ventricular Tachycardia in Sisters Initially Diagnosed With Long QT Syndrome

**DOI:** 10.1016/j.jaccas.2026.108044

**Published:** 2026-06-03

**Authors:** Christine Zhang, Abdullah Sarkar, Eugene Wong, Marmar Vaseghi, Jessica J. Wang

**Affiliations:** a Department of Integrative Biology and Physiology, University of California-Los Angeles, Los Angeles, California, USA; b Division of Cardiology, Department of Medicine, David Geffen School of Medicine at University of California-Los Angeles, Los Angeles, California, USA; c Division of Clinical Genetics, Department of Human Genetics, University of California-Los Angeles, Los Angeles, California, USA; d Institute for Precision Health, University of California-Los Angeles, Los Angeles, California, USA

**Keywords:** genetic disorders, genetics, ventricular tachycardia

## Abstract

**Background:**

The QT interval is often prolonged in the immediate postcardiac arrest period, but not all patients with a prolonged QT interval have long QT syndrome (LQTS).^1^ Re-evaluation of probands with presumed LQTS with a broader gene panel can correct a misdiagnosis, provide clarity in management, and enhance family counseling and screening.

**Case Summary:**

A 29-year-old female was diagnosed with LQTS in childhood based on borderline QTc interval and a family history of a sister with presumed LQTS after cardiac arrest. Updated genetic testing in the proband revealed a likely pathogenic *RYR2* variant and a benign *KCNE1* variant. Familial testing confirmed that the patient carried the *RYR2* variant, establishing a diagnosis of catecholaminergic polymorphic ventricular tachycardia.

**Discussion:**

This case underscores the value of confirming the underlying molecular diagnosis in determining arrhythmic risk and guiding family evaluation. Broader or updated genetic testing can clarify disease mechanism, prevent misdirected management, and support targeted risk assessment in relatives.

**Take-Home Messages:**

The differential diagnosis of cardiac arrest is broad, including arrhythmia syndromes, arrhythmogenic cardiomyopathy, and nongenetic causes. Comprehensive genetic testing and counseling are essential for informing management of sudden cardiac arrest survivors and their relatives.

## Visual Summary

**Figure:**
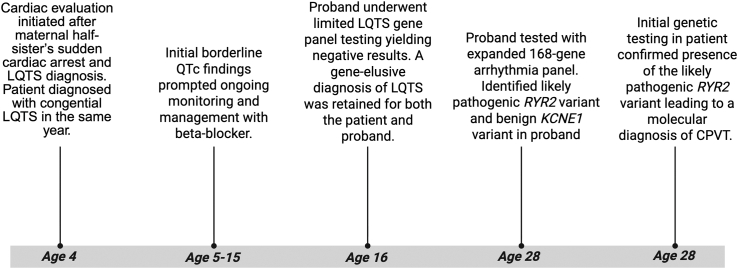

## History of Presentation

A 29-year-old female was referred to the cardiovascular genetics clinic for reevaluation of a childhood diagnosis of long QT syndrome (LQTS). She had remained clinically stable on beta-blocker therapy, with no history of syncope, chest pain, or palpitations in adulthood.

## Past Medical History

**Figure 1:**
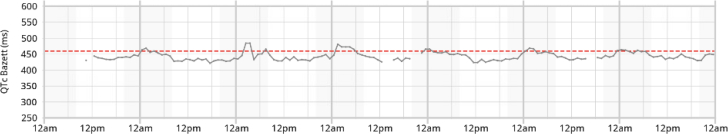
Extended Holter Monitor QT Analysis (Age 28) Seven-day Holter monitoring (6 days 20 hours analyzable) demonstrated sinus rhythm with an average heart rate of 77 beats/min. Mean QT was 398 ms, and maximal QT was 435 ms, both within the normal range.

The patient was initially evaluated in childhood for possible congenital LQTS after her maternal half-sister (the proband) was clinically diagnosed with LQTS following a sudden cardiac arrest with ventricular fibrillation while swimming at age 13. The patient underwent a 24-hour pediatric Holter monitor at age 4, which, according to the pediatric cardiologist's clinical documentation, showed borderline QT prolongation with 38.7% of beats demonstrating a QTc >450 ms. At age 8, repeat 24-hour Holter monitoring revealed a mean QTc of 442 ms, with 36% of beats exceeding an interval of 450 ms. Given her persistently borderline QTc interval and her half-sister's diagnosis, the patient was started on propranolol 60 mg daily at the age of 9 for possible LQTS. Over the next several years, the patient continued beta-blocker therapy and remained clinically stable, staying physically active and exercising without any events. Her annual follow-ups, including echocardiograms (ECGs), Holters, and exercise stress testing, showed no significant arrhythmias or worsening QT intervals. At age 16, she experienced an episode of presyncope, in the setting of outdoor sports and high temperatures, which was attributed to be vasovagal in nature. By age 18, the patient changed beta blockers to atenolol 100 mg daily given reports of neuropsychiatric symptoms, which improved tolerability. Subsequent treadmill stress tests between ages 18 and 21 years continued to show adequate beta-blockade, occasional premature ventricular contractions during peak exertion, and good exercise tolerance. Holter monitors and ECGs remained unremarkable (Figure 1), and the patient reported no syncope, chest pain, or palpitations throughout this period.

**Figure 2:**
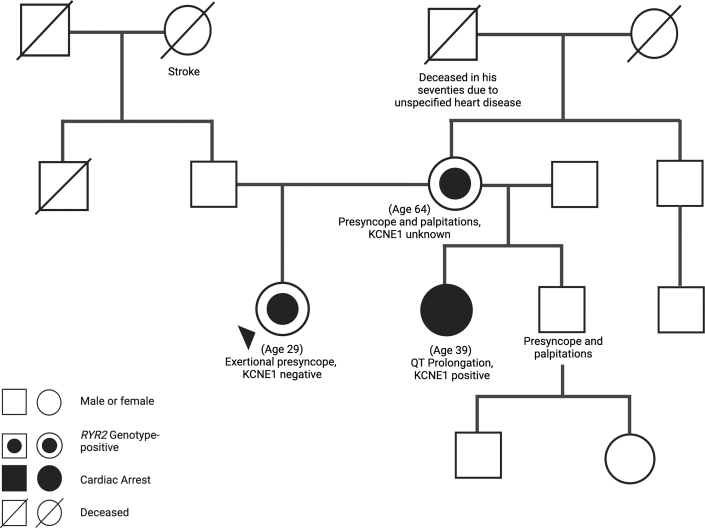
Family Pedigree Demonstrating Maternal-Familial Clustering of Arrhythmic Symptoms and *RYR2* Variant Carriers The pedigree shows a maternal-familial clustering pattern of presyncope, palpitations, and cardiac events. The proband experienced cardiac arrest at age 13 and is genotype-positive for a pathogenic *RYR2* variant, as well as a benign KCNE1 variant. The patient is genotype-positive for the same *RYR2* variant and had a single episode of exertional syncope at age 16 but has remained otherwise asymptomatic. The patient's maternal half-brother has a history of presyncope and palpitations, although the age of onset is unknown. The patient's mother is depicted as an obligate carrier based on inheritance. Stroke and unspecified cardiac disease are noted in earlier generations.

As mentioned earlier, the family history is significant for sudden cardiac arrest in the patient's maternal half-sister at age 13, with the diagnosis of LQTS being attributed to hospital ECG demonstrating sinus rhythm with a QTc of 464 ms. The maternal half-brother was reported to have presyncopal episodes and palpitations, but has not undergone formal cardiac evaluation. The patient's mother has a history of syncope, and given the proband and patient's diagnosis, she was also diagnosed with LQTS; prior resting ECG showed sinus bradycardia with a QT interval of 436 ms while on beta-blocker therapy, with rare premature atrial contractions and premature ventricular contractions on stress testing. The maternal grandfather had childhood syncopal episodes and died of unspecified heart disease in his seventies (Figure 2).

## Differential Diagnosis

The differential diagnosis of cardiac arrest is broad, including arrhythmia syndromes, arrhythmogenic cardiomyopathy, and nongenetic causes. Maternal half-sister's ventricular fibrillation arrest in the setting of borderline QT interval prompted a re-assessment of the prior diagnosis of LQTS.

## Investigations

Maternal half-sister's initial genetic testing in 2012 included a targeted multigene LQTS panel, which was negative for any pathogenic variants. As the patient herself had an equivocal phenotype, it was recommended that her half-sister, the most definitely affected individual, update her genetic testing. The half-sister underwent testing via a 168-gene arrhythmia and cardiomyopathy panel through a commercial testing laboratory, identifying a heterozygous likely pathogenic *RYR2* c.1199A>G (p.Asp400Gly) variant, associated with autosomal dominant catecholaminergic polymorphic ventricular tachycardia (CPVT), a heterozygous *KCNE1* c.253G>A (p.Asp85Asn, D85N) polymorphism for acquired LQTS, and variants of uncertain significance in *ANK2*, *SLC2A1*, and *TNNT2* genes. Following these findings, the patient underwent familial variant testing for *RYR2* and *KCNE1* and was confirmed to carry the familial *RYR2* variant, without the *KCNE1* risk allele. This established a revised molecular diagnosis of *RYR2*-related CPVT in the patient, explaining her normal resting QTc and occasional exertional ectopy in the absence of structural heart disease.

## Management

As the patient continues to remain at risk of exertional arrhythmias in the setting of CPVT, therapy with atenolol was continued.

## Outcome and Follow-Up

The patient remains clinically stable without syncope, sustained arrhythmias, or other cardiac symptoms. The identification of a likely pathogenic RYR2 variant clarified the underlying diagnosis and arrhythmic risk profile, supporting continued beta-blocker therapy and longitudinal follow-up. She remains under regular surveillance with cardiac electrophysiology.

## Discussion

This case illustrates the need for caution when designating a diagnosis of LQTS, particularly in the setting of cardiac arrest, where transient QT prolongation is common and the underlying etiology is often unclear. Overreliance on early postarrest ECG findings without comprehensive follow-up can result in a premature diagnosis of LQTS, potentially leading to inadequate management. Although both genetic conditions are commonly treated with beta-blockers, CPVT often requires additional therapies, including flecainide, implantable cardioverter-defibrillator therapy, or left cardiac sympathetic denervation.^1^^,^^2^ Longitudinal follow-up in our patient consistently showed normal QTc and exertion-induced symptoms without structural heart disease, making it challenging to assign a cardiac diagnosis to her in spite of a concerning family history. Given a lack of a specific phenotype seen in our patient, updated genetic testing was recommended for her maternal half-sister, who is clearly affected, ultimately yielding a heterozygous likely pathogenic *RYR2* variant (p.Asp400Gly) consistent with a molecular diagnosis of autosomal dominant CPVT. Cascade genetic testing in our patient enabled clarification of the diagnosis, appropriate monitoring, and informed genetic counseling for reproductive decision-making.

Pathogenic variants in the *RYR2* gene are the most frequent cause of CPVT and are associated with stress-induced bidirectional or polymorphic ventricular tachycardia in the absence of structural cardiac abnormalities.^3^ Notably, the presence of a *KCNE1* D85N polymorphism in the patient's half-sister, but not in our patient, may account for phenotypic variability. The *KCNE1* D85N is a risk allele present in 1% and 2% of the general population and acquired LQTS.^4^ In the patient's sister, this variant may have contributed to QT prolongation and a misdiagnosis of LQTS. In addition, the variant may act as a phenotypic enhancer in the proband, resulting in prolonged QT interval which may heighten vulnerability to arrhythmia during adrenergic stress, such as during physical activity. Its absence in our patient may partially account for her milder CPVT phenotype, despite her sharing the same primary *RYR2* variant. Information on potential genetic modifiers is critical in the discussion of individual risks among relatives sharing the same primary pathogenic variant within a given family.

Genetic testing is increasingly considered for unexplained cardiac arrest survivors, though guidelines often prioritize it only after a suspected phenotype is identified.^5^^,^^6^ Genetic studies have shown that pathogenic variants in arrhythmia and cardiomyopathy genes can be found in the absence of pathognomonic clinical findings,^7^ highlighting the importance of clinical phenotyping, family history, and comprehensive genetic evaluation.

## Conclusions

This case highlights the utility of broad multigene genetic testing in distinguishing between overlapping arrhythmic phenotypes when the differential diagnosis is uncertain.^8^ Broad multigene genetic testing clarified the underlying etiology, reclassifying the patient and her family's condition as CPVT, thereby affirming the appropriate course of management. Establishing a molecular diagnosis also enabled accurate risk assessment and counseling for unaffected relatives.

**Visual Summary:**
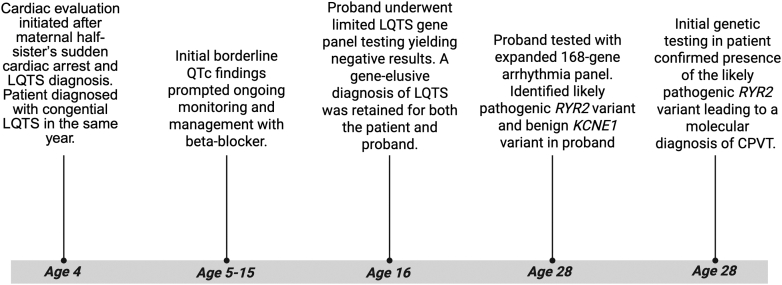
Timeline of Clinical Case

## Funding Support and Author Disclosures

The authors have reported that they have no relationships relevant to the contents of this paper to disclose.

**Take-Home Messages**

• The differential diagnosis of cardiac arrest is broad, spanning from arrhythmia syndromes to arrhythmogenic cardiomyopathy and nongenetic causes.
• Comprehensive genetic testing and molecular diagnosis are essential to guide accurate familial risk screening, assessment, and management in sudden cardiac death survivors.
